# Correlation between α1-Antitrypsin Deficiency and SARS-CoV-2 Infection: Epidemiological Data and Pathogenetic Hypotheses

**DOI:** 10.3390/jcm10194493

**Published:** 2021-09-29

**Authors:** Andrea Vianello, Gabriella Guarnieri, Fausto Braccioni, Beatrice Molena, Sara Lococo, Alessia Achille, Federico Lionello, Leonardo Salviati, Marco Caminati, Gianenrico Senna

**Affiliations:** 1 Department of Cardiac Thoracic Vascular Sciences and Public Health, University of Padova, 35122 Padova, Italy; gabriella.guarnieri@unipd.it (G.G.); fausto.braccioni@aopd.veneto.it (F.B.); beatrice.molena@unipd.it (B.M.); sara.lococo@aopd.veneto.it (S.L.); alessia.achille@aopd.veneto.it (A.A.); federico.lionello@aopd.veneto.it (F.L.); 2 Department of Pediatrics, University of Padova, 35122 Padova, Italy; leonardo.salviati@unipd.it; 3 Asthma Center and Allergy Unit, University of Verona, 37129 Verona, Italy; marco.caminati@univr.it (M.C.); gianenrico.senna_01@univr.it (G.S.)

**Keywords:** α1-antitrypsin deficiency, COVID-19, SARS-CoV-2, genetics

## Abstract

The most common hereditary disorder in adults, α1-antitrypsin deficiency (AATD), is characterized by reduced plasma levels or the abnormal functioning of α1-antitrypsin (AAT), a major human blood serine protease inhibitor, which is encoded by the SERine Protein INhibitor-A1 (*SERPINA1*) gene and produced in the liver. Recently, it has been hypothesized that the geographic differences in COVID-19 infection and fatality rates may be partially explained by ethnic differences in *SERPINA1* allele frequencies. In our review, we examined epidemiological data on the correlation between the distribution of AATD, SARS-CoV-2 infection, and COVID-19 mortality rates. Moreover, we described shared pathogenetic pathways that may provide a theoretical basis for our epidemiological findings. We also considered the potential use of AAT augmentation therapy in patients with COVID-19.

## 1. Introduction

Severe acute respiratory syndrome coronavirus 2 (SARS-CoV-2), which originated in Wuhan, China, has resulted in a pandemic, with more than 183 million confirmed cases and approximately 4 million deaths (as of 30 June 2021) [1]. According to the worldwide epidemiologic data, there are remarkably different infection and mortality rates for SARS-CoV-2 between different countries; the numbers of deaths per million of the population in Europe and the Americas were approximately 43 and 21 times higher than those in East Asia, respectively (as of 25 February 2021) [2]. Moreover, some concerns remain regarding the great interindividual differences in the clinical severity of coronavirus disease 2019 (COVID-19), which cannot be completely explained by environmental factors, comorbidities, and age-related fragility [3].

On the basis of these simple observations and the susceptibility of hosts as the main infection component, it could be argued that genetic differences among populations, ethnicities, and individuals may contribute to the different epidemiological and clinical manifestations of COVID-19 [2,4].

Recent studies have investigated genetic susceptibility to SARS-CoV-2 and reported that genetic errors and gene loci are associated with approximately 20% of life-threatening COVID-19 cases, most of which are involved in two immune signaling pathways—i.e., the interferon-mediated antiviral signaling and chemokine-mediated inflammatory signaling pathways [5]. Interestingly, Souilmi et al. showed a strong genomic signature in the East Asian population, likely due to ancient coronavirus epidemics that may have emerged more than 20,000 years ago, involving gene variants encoding virus-interacting proteins (VIPs), which may imply a difference in genetic susceptibility to SARS-CoV-2 infection [6].

In patients with COVID-19, disease severity and high mortality are associated with respiratory failure due to acute respiratory distress syndrome (ARDS) and multiple organ failure due to an uncontrolled cytokine storm, with significantly increased levels of several inflammatory mediators, including IL-6, interferon gamma (INF-γ), Tumor Necrosis Factor alpha (TNF-α), interleukin 17 (IL-17), and IL-8 [3].

Therefore, we focus on the possible role of α1-antitrypsin deficiency (AATD), an inherited disorder known for nearly 60 years [7], in the vulnerability of some individuals to SARS-CoV-2 infection and severe COVID-19 progression. Indeed, α1-antitrypsin (AAT) inhibits neutrophil elastase (NE), which, when unopposed, can cleave many of the structural proteins of the lung as well as innate immune proteins. The anti inflammatory and tissue-protective properties of AAT are underscored by several studies suggesting its usefulness in transplantation medicine [8].

Moreover, we report epidemiological data on the distribution of both conditions and describe common pathogenic pathways that may support the importance of AAT in antagonizing the SARS-CoV-2 infection.

## 2. Genetics, Epidemiology, and Clinical Relevance of AATD

AAT, encoded by the *SERPINA1* gene, is an acute-phase glycoprotein that is mainly synthesized and secreted by hepatocytes and is the main serum protease inhibitor. However, other cell types can also express this protein. A study by Van’t Wout et al. demonstrated that AAT is produced by differentiated monocyte-derived macrophages, monocyte-derived dendritic cells, and alveolar macrophages [9]. NE, an enzyme responsible for the proteolytic degradation of lung elastase and alveolar and interstitial lung tissue, and AAT, which primarily acts as an inhibitor of the serine protease, act in concert to maintain protease–anti-protease homeostasis, thus protecting the lung tissue from proteolytic digestion [10]. AAT also has anti-coagulation effects and can protect against inflammation, cell death, and the formation of neutrophil extracellular traps (NETs) [11]. Notably, AAT has the capacity to bind and inhibit the SARS-CoV-2-priming protease, transmembrane serine protease 2 (TMPRSS2), on host cells, thus inhibiting the first step in the SARS-CoV-2 cell cycle [12].

Over 120 mutations in the *SERPINA1* gene encoding AAT have been identified, of which the most common are the Z and S mutations, which are characterized by the substitution of glutamic acid with lysine or valine at positions 342 and 264 of the polypeptide, respectively, as well as by reduced circulating protein levels [13]. Although AATD has long been considered a rare disease, it is now identified as a prevalent hereditary disease among Caucasian individuals in Northern Europe and immigrants from these countries to the New World. Nevertheless, it remains highly underdiagnosed due to its clinical overlap with other pulmonary disorders, including chronic obstructive pulmonary disease (COPD) and severe asthma [14,15]. The highest prevalence of the PI*Z variant was recorded in Northern and Western European countries (mean gene frequency: 0.0140), peaking in southern Scandinavia, Denmark, the Netherlands, the UK, and northern France (gene frequency > 0.0200) [16]. The PI*SZ genotype typically exhibits an AAT level less than half that of a normal (PI*MM) individual and shows a similar lung disease risk between PI*MM and PI*ZZ patients, given the same level of smoke exposure. Epidemiological studies have estimated that there are over 500,000 PI*SZ individuals in Europe [17].

In a worldwide analysis of data from 514 cohorts (69 countries across 11 geographical areas), De Serres estimated the prevalence of PI*ZZ, PI*SZ, and PI*SS individuals to be 3.4 million [16]. Extrapolating this to the world population in 2015, the prevalence of AATD individuals was estimated to be 5.64 million [18].

Clinically, patients with AATD with a homozygous PI*ZZ genotype have an increased risk of developing COPD (including chronic bronchitis, emphysema, and bronchiectasis) and chronic liver disease, an increased susceptibility to viral infection, and a prolonged course of infection [19,20,21,22]. Several multifactorial elements may contribute to the onset and severity of clinical manifestations, including smoking, alcohol intake, air pollutants, and/or passive smoke exposure [19].

Once COPD has been diagnosed, patients with AATD should receive lifestyle suggestions and pharmacological treatment according to standard recommendations [22]. Moreover, patients should be referred to an expert center, where indications for augmentation therapy should be evaluated. AAT augmentation therapy is a disease-specific treatment aimed at restoring protective levels of the deficient enzyme [10]. In line with the COPD treatment guidelines, pulmonary rehabilitation, lung volume reduction, and lung transplantation should also be considered for the management of patients with AATD at the advanced stage of the disease [23,24].

## 3. AATD and COVID-19: Geographical Overlap and Data from Clinical Registries

During the COVID-19 pandemic, several epidemiologic studies have reported that the geographical distribution of patients with AATD is similar to that of confirmed cases of COVID-19. Of importance, most studies have methodological limitations, including non-official data sources, unknown sample sizes, and different techniques for ascertaining AAT variants, leading to the potential underestimation of the prevalence of AATD [16].

In April 2020, a geographical overlap between the prevalence of allelic variants of *SERPINA1* and severe cases of COVID-19 was reported in Italy [25]. In parallel with the distribution of AAT mutations following a north-to-south gradient, with 47% of the total number registered in the Lombardia region (237 out of 508) [26], an inhomogeneous distribution of COVID-19 cases was reported, with 85% of the total fatal cases registered in northern Italy (51.9% in the Lombardia region in particular) and an abrupt decrease from north to south. The AATD population size is much smaller than the COVID-19 patient population, but it should be pointed out that AATD is largely under-recognized in Italy, with a relationship between diagnosed and expected cases of 1 to 460. In particular, one carrier or deficiency-allele combination phenotype is expected for every 13.6 individuals, with greater frequencies in PI*MS and PI*MZ genotypes [16]. Recent data have confirmed a north-to-south gradient of COVID-19 mortality in Italy, with a decrease from 236–378 deaths/100,000 inhabitants in the northern regions to 65 deaths/100,000 inhabitants in southern Italy (Calabria region) [1].

Two later studies confirmed a correlation between the COVID-19 pandemic and the prevalence of AATD on a global scale. In September 2020, Yoshikura reported that the prevalence of AATD was correlated with the epidemiologic distribution of COVID-19, particularly in countries with a high AATD prevalence, showing an epidemic curve characterized by an initial rise, followed by a prolonged duration of high infection and mortality rates, whereas countries with a low AATD prevalence showed a more rapid decrease in mortality rate after the initial rise [27]. Shapira et al. observed that Pi*Z alleles were 8-fold less frequent in Middle East/Far East countries (2:1000 individuals) than in South European countries (17:1000 individuals). This difference was more remarkable (17-fold) when the Pi*S allele was considered, as the prevalence in Far East countries was 5:1000 compared to 86:1000 in South European countries [28]. In addition, a large difference in mortality rates was found between Middle East/Far East and South European countries, such as Thailand, Vietnam, and Cambodia, accounting for 5, 0.34, and 6.9 deaths per 100,000 individuals, respectively, compared with a mortality rate of 216, 174, and 123 per 100,000 individuals in Italy, Spain, and Greece, respectively. The authors also found a significant correlation (R = 0.54; *p* = 0.00000198) between the frequency of Pi*Z and Pi*S alleles and mortality rates due to COVID-19 in 67 countries. The correlation was still significant after correcting for confounding factors, including urbanization and age distribution. Although these data are suggestive, the authors underline that additional confounding factors should be considered, including the different control measures established by governments, socioeconomic status, and population health [28].

According to epidemiological data, Bhattacharyya et al. observed that within 3 months of the onset of the COVID-19 pandemic, SARS-CoV-2 evolved into 10 additional subtypes, and it was found that the mutant subtype (614G) outcompeted the pre-existing type (614D) significantly faster in Europe and North America than in East Asia. The G mutant is characterized by a novel NE cleavage site near the S1-S2 junction of the spike protein. The authors hypothesized that the elevation of the NE level at the site of infection might have enhanced the activation of the spike protein, thus facilitating host cell entry for 614G. Therefore, AATD could promote the host cell entry of the 614G virus by retarding the inhibition of NE and consequently enhancing the activation of the spike protein. Provided that AATD is more prevalent in European and North American populations, this could explain why the SARS-CoV-2 subtype 614G spread more easily in these geographical regions than in East Asia [29].

Ferrarotti et al. recently investigated whether patients with severe AATD had an increased risk of severe COVID-19 infection. Therefore, they collected data on COVID-19 symptoms, laboratory-confirmed infection, hospitalization, and treatment directly administered to Italian severe AATD subjects using a telephone survey and compared their findings with data collected by the Istituto Superiore di Sanità on the total population in Italy. The authors found a higher frequency of SARS-CoV-2 infection in the AATD cohort (3.8%) compared to the national data regarding infection, and patients with severe AATD had a relative risk of 8.8 (95% confidence interval (CI): 5.1–20.0; *p* < 0.0001) for symptomatic SARS-CoV-2 infection. Moreover, the relative risk (RR) was higher in patients with AATD and pre-existing lung diseases (RR 13.9; 95% CI: 8.0–33.6; *p* < 0.001) [30].

Faria et al. collected data from all patients with AATD at the Pulmonology Department in a tertiary hospital in Porto (Portugal) and compared patients with AATD who were diagnosed with COVID-19 with the remaining AATD cohort based on patients’ pre-infection AAT serum levels, clinical, and functional status. The Pi*ZZ genotype was significantly associated with greater COVID-19 incidence (33.3%, *p* = 0.012), followed by Pi*MS (14.3%) and Pi*SZ (10.0%). Moreover, the baseline AAT levels were significantly lower in patients with COVID-19 (42.9 ± 12.1 vs. 65.5 ± 2.8 mg/dL; *p* = 0.012) [31].

Unlike previous findings, Schneider and Strnad recently assessed the association between AATD and COVID-19 in the United Kingdom Biobank, a community-based cohort with >500,000 participants, and showed that the most common and mild AATD genotypes were not associated with increased SARS-CoV-2 infection rates or increased SARS-CoV-2 fatalities, whereas the number of severe AATD cases was too low to allow definitive conclusions to be drawn [32].

In conclusion, most epidemiological observations agree that there is a correlation between the SARS-CoV-2 epidemic, the clinical severity of COVID-19, and the prevalence of AATD.

Results from epidemiologic studies are summarized in Table 1.

## 4. Shared Pathogenic Pathways

To provide a theoretical basis for emerging epidemiological data, several studies have focused on the possibility of shared pathogenic pathways between AATD and SARS-CoV-2 infection.

Indeed, AAT has several biological functions that may antagonize SARS-CoV-2 infection and pathophysiologic processes resulting in cellular entry. According to Bai et al., the potential AAT protective mechanisms of action can be summarized as follows [33]:

1. Enhancement of host immunity: The antiviral effects of AAT have been documented for different RNA viruses, including the influenza virus [34] and human immunodeficiency virus (HIV) [35,36,37,38,39]. Indeed, AAT has been proven to block HIV entry into CD4^+^ lymphocytes and inhibit HIV replication. Moreover, AAT has been shown to enhance host immunity against Pseudomonas aeruginosa [40] and Mycobacterium intracellulare [41] by inducing autophagy, which is also implicated in the control of MERS-CoV infection [42].
2. Inhibition of TMPRSS2: SARS-CoV-2 is an enveloped, single-stranded RNA virus. A pivotal role in cell entry is played by a viral surface “spike” protein, which is arranged in a trimeric form. The spike protein is composed of two subunits, S1 and S2. Two sequential cleavages are needed for the virus to enter the human cells. The first is performed by furin or furin-like proteases (which are ubiquitous in the human body) at the S1/S2 site. Subsequently, the spike protein binds to the host cell surface receptor angiotensin-converting enzyme 2 (ACE2) through its receptor-binding domain, undergoing conformational changes that make it possible for a second cleavage mediated by transmembrane serine protease 2 (TMPRSS2), commonly expressed in epithelial cells [43]. Following cleavage, SARS-CoV-2 may enter human cells through endosomal and/or non-endosomal pathways. In the first case, the virus enters the endosomes, whereas in the second case, the envelope directly fuses with the cell plasma membrane. In addition to furin/furin-like proteases and TMPRSS2, other proteases are involved in SARS-CoV-2 cell entry, which enhance the infectivity and transmissibility of the virus. In particular, several membrane-associated serine proteinases, including proprotein convertase 1 (PC1), trypsin, and matriptase-2, may synergize with or replace TMPRSS2 as the cellular activator of SARS-CoV-2 [44]. ACE2 is a component of the renin-angiotensin system that plays a role in the systemic regulation of the cardiovascular and renal systems, lungs, and liver by acting on blood pressure, electrolyte balance control mechanisms, and inflammation. ACE2 also plays a protective role against lung injury, diabetic cardiovascular complications, myocardial infarction, and disseminated intravascular coagulation. Interestingly, all of these conditions are associated with severe COVID-19 outcomes [45]. In vitro, TMPRSS2 inhibition has been demonstrated to prevent SARS-CoV-2 cell entry [46,47,48]. Based on the demonstration of the in vitro inhibition of SARS-CoV-2 cell entry by AAT [49,50], Wettstein et al. hypothesized that its effect could be related to the inhibition of TMPRSS2-mediated priming [50].
3. Anti-inflammatory activity: AAT has strong anti-inflammatory properties, including the following:
  - Inhibition of pro-inflammatory transcription factor NFκB [39,51,52];
  - Binding of extracellular IL-8 [53] with attenuated Akt activation [54], resulting in a decreased risk of acute lung injury [55];
  - Inhibition of neutrophil superoxide synthesis [56] and reduction in oxidative stress levels that are commonly increased in COVID-19 [57];
  - Inhibition of disintegrin/metalloproteinase 17 (ADAM17) [45]: ADAM17 is activated by the spike protein of coronaviruses and cleaves membrane-bound TNF-α to soluble TNF-α. Moreover, ADAM17 causes ACE2 shedding [58]. ACE2 shedding may increase inflammatory response by preventing the formation of the anti-inflammatory peptides, angiotensin-(1–7) and angiotensin-(1–9) [59].
4. Protection against acute lung injury (ALI): AAT inhibits NE activity, which is known to mediate ALI at the sites of acute inflammation by inducing the release of IL-8 from neutrophil vesicles and facilitating the conversion of pro-IL-1β to IL-1β [60,61]. Moreover, AAT prevents ACE2 shedding by inhibiting ADAM17; increased ACE2 levels may inactivate bradykinin, which is essential for the leakage of exudate through the alveolar-capillary membrane in patients with non-cardiogenic pulmonary oedema [62].
5. Inhibitory effect on thrombin and delayed thrombus formation: AAT antagonizes thrombin, a serine protease [57], and inhibits other pro-coagulant proteins, thus contributing to the delay of micro- and macro-thrombi formation [57], which is a common event in COVID-19 [57,62,63].
6. Inhibition of NETs adherence: NETs essentially consist of neutrophil-derived decondensed chromatin (cell-free DNA) combined with other proteins (i.e., elastase and cathepsin G), aimed at trapping and killing extracellular pathogens. In patients with COVID-19, the aberrant production of NETs plays a pathogenic role in immuno-thrombosis, mucous secretion, and cytokine production [64,65,66,67,68,69]. AAT may inhibit elastase, which is crucial for NET formation. Ex vivo studies have shown that AAT modifies the shape of NETs and reduces their adherence [70].
7. Protection against endothelial cell apoptosis: AAT inhibits caspase-3 in endothelial cells [71], an executioner caspase in the classical apoptotic pathway, thereby antagonizing endothelial injury [71,72]. AAT also decreases oxidative stress, inflammation, and cell wall deterioration [71].

**Figure 1:**
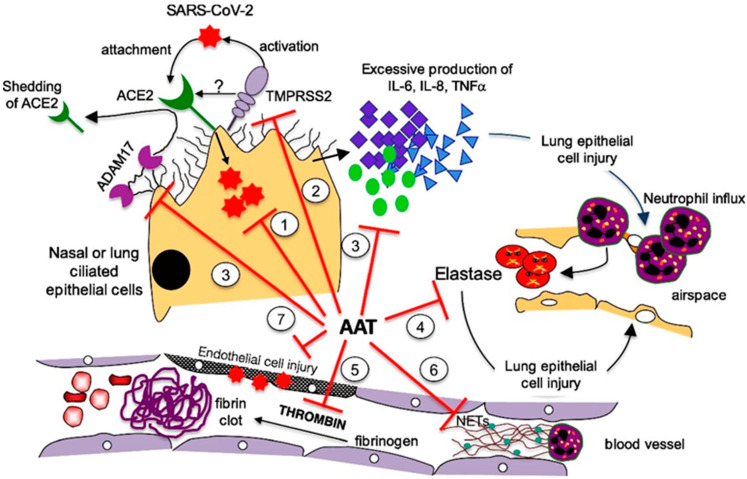
Shared pathogenic pathways between AATD and SARS-CoV-2 infection (1 = host immune dysregulation; 2 = increased activation of TMPRSS2; 3 = increased neutrophils mobilization; 4 = irrelevant inhibition of NE activity; 5 = reduced clotting antagonization; 6 = elevated NETs formation; 7 = increased endothelial cell apoptosis). (ACE2 = angiotensin-converting enzyme 2; IL-6 = interleukin 6; IL-8 = interleukin 8; NETs = neutrophil extracellular traps; TMPRSS2 = transmembrane serine protease 2; TNF-*α* = tumor necrosis factor-alpha) [33].

In view of this, shared pathogenic pathways can be hypothesized between AATD and SARS-CoV-2 infection; these are displayed in Figure 1.

To identify immune factors of the respiratory tract that may be effective against SARS-CoV-2, Wettstein et al. created and screened two peptide/protein libraries derived from homogenized human lung and bronchoalveolar lavage for the inhibitors of SARS-CoV-2 spike-driven entry. The analysis of the antiviral fractions revealed the presence of AAT, which inhibits SARS-CoV-2 entry at physiological concentrations and suppresses viral replication in cell lines and primary cells, including human airway epithelial cultures. They also demonstrated that AAT binds and inactivates the serine protease TMPRSS2 and concluded that AAT plays an important role in the innate immune defense against the novel coronavirus [49].

In line with these observations, Yang et al. suggested that patients with AATD should be considered to be at an increased risk for SARS-CoV-2 infection and the development of severe COVID-19 based on the following assumptions:

1. Reduced levels of functional AAT would prompt the activation of TMPRSS2, thus promoting SARS-CoV-2 cell entry;
2. The lack of thrombin and plasmin inhibition would increase the risk of coagulation disorders;
3. Reduced anti-inflammatory, anti-cell death, anti-protease, and anti-coagulation activities would result in a greater probability of developing severe ALI [73].

Shimi et al. hypothesized that low vitamin D levels in patients with COVID-19 could result in acquired AATD, leading to greater clinical severity and a higher risk of death [74].

From a clinical point of view, it should be noted that in patients with COVID-19 who were admitted to the intensive care unit (ICU), higher ratios of interleukin (IL) 6 to α1-antitrypsin predicted a prolonged ICU stay and higher mortality, whereas lower ratios of IL-6 to α1-antitrypsin were associated with clinical resolution [3].

## 5. Augmentation Therapy for Patients with COVID-19

To date, AAT supplement therapy has been largely used to slow disease progression and reduce exacerbations in patients with AATD and COPD. The multiple AAT activities discussed above have suggested its potential therapeutic use for patients with COVID-19. The biological functions of AAT that may antagonize SARS-CoV-2 infection are outlined in Table 2.

Three clinical trials have been undertaken to examine the potential benefits of α1-proteinase inhibitors in patients with COVID-19 [75,76,77]; however, results were not available at the time of this review. To date, only sporadic case reports and short case series have been published concerning the possible role of AAT as a treatment option for COVID-19.

AAT was successfully used in a 43-year-old patient with COVID-19 who was affected with cystic fibrosis and placed on a waiting list for lung transplantation. She developed severe ARDS and required mechanical ventilation (MV). Poor prognosis and severe inflammatory phenotype prompted physicians to administer AAT as an off-label therapy at 120 mg/kg/week for four consecutive weeks. Rapid clinical, laboratory, and radiographic improvement was detected, and the patient was liberated from MV and discharged from the hospital [78].

Ritzmann et al. administered inhaled or combined inhaled/intravenous AAT in nine patients with mild-to-moderate COVID-19. After the first AAT administration, the C-reactive protein (CRP) levels abruptly decreased. All patients were discharged from the hospital approximately 20 days after admission. Three out of nine patients in a matched control group died and CRP decrease was delayed [79].

Finally, Martini et al. indicated that AAT treatment via aerosol by humid heat vaporization (40–41 °C) should be considered in the early phase of COVID-19 to hamper SARS-CoV-2 entry into the host cells [80].

When advocating for their use in patients with COVID-19, it should be emphasized that AAT products have been marketed for many decades and found to be safe. Moreover, treatment protocols and schedules are largely available.

Based on multiple early reports of patients admitted to the hospital with COVID-19 showing that subjects with chronic obstructive respiratory disease were significantly under-represented in these cohorts, therapeutic options commonly used in patients with AATD, including bronchodilators (long-acting muscarinic antagonist, long-acting beta-2 agonists, etc.) and inhaled corticosteroids, have been tested aiming to establish whether they would be an effective treatment for early COVID-19. In this regard, a study by Ramakrishnan et al. showed that the early administration of inhaled budesonide can reduce the likelihood of needing urgent medical care and decrease time to recovery after early COVID-19 [81].

## 6. Conclusions

As AATD represents a large population in geographic areas with a high incidence of COVID-19 and high mortality associated with the disease, understanding the interrelation between these conditions is essential in order to reduce COVID-19-related morbidity and mortality. The potential beneficial effects of AAT make it an ideal candidate for COVID-19 treatment; however, in vivo studies with animal models and clinical trials are needed to validate its large-scale utilization.

## Figures and Tables

**Table 1:** Main results from epidemiologic studies on the overlap between α1-antitrypsin deficiency and SARS-CoV-2 infection (NA = not available).

Authors	Geographical Area	Population Size	Main Findings
Vianello et al. [25]	Italy	60,461,826	Overlap between variants of the *SERPINA1* gene and severe cases of COVID-19
Yoshikura [28]	Global scale	7.9 billion	Prevalence of AATD correlates with distribution of COVID-19
Shapira et al. [29]	Global scale	NA	Correlation between AATD prevalence and COVID-19 mortality rates between Middle East/Far East and South Europe
Bhattacharyya et al. [30]	Europe, North America	NA	Increased risk of SARS-CoV-2 subtype 614G infection explained by higher AATD prevalence
Ferrarotti et al. [31]	Italy	209	Higher frequency of SARS-CoV-2 infection in AATD cohort compared to national data
Faria et al. [32]	Portugal	77	PiZZ genotype associated with greater COVID-19 incidence
Schneider et al. [33]	United Kingdom	500,000	Mild AATD genotypes not associated with increased SARS-CoV-2 infection or fatality rates

**Table 2:** Protective effects of augmentation therapy in SARS-CoV-2 infection.

Protective Effect	Underlying Mechanism
Antiviral	Enhancement of host immunity
	Inhibition of TMPRSS2
Anti-inflammatory	Reduced IL-8 release; IL-8 binding
	Inhibition of NFκB and ADAM17
Prevention of acute lung injury	Inhibition of NE and ADAM17
Prevention of thromboembolism	Thrombin antagonization
	Inhibition of NET adherence
Prevention of endothelial cell injury	Inhibition of caspase-3

(ADAM17 = disintegrin/metalloproteinase 17; IL-8 = interleukin 8; NE = neutrophil elastase; NET = neutrophil extracellular trap; NFκB = nuclear factor-kappa B; TMPRSS2 = transmembrane serine protease 2).

## Data Availability

Not applicable.
